# Understanding lightbulb moments: Meaning-making in visual morphology from comics and emoji

**DOI:** 10.3758/s13421-025-01734-9

**Published:** 2025-06-06

**Authors:** Lenneke Doris Lichtenberg, Bien Klomberg, Joost Schilperoord, Neil Cohn

**Affiliations:** Department of Communication and Cognition, Tilburg School of Humanities and Digital Sciences, Tilburg Center for Cognition and Communication, Tilburg University, P.O. Box 90153, 5000 LE Tilburg, The Netherlands

**Keywords:** Visual morphology, Visual metaphors, Upfixes, Cross-modal priming

## Abstract

How do we interpret a lightbulb above a head in visual images to mean inspiration? We investigated the semantic processing of these “upfixes” like lightbulbs or gears that float above characters' heads. We examined the congruity of face-upfix dyads presented sequentially with words describing their literal (“lightbulb”) or non-literal meanings (“inspiration”). To examine if upfixes alone sponsor meanings, we showed participants upfixes that either matched or mismatched the facial expression (e.g., lightbulb over an excited vs. sad face). Literal words always evoked faster response times for face-upfix dyads when presented before the images. When images appeared before words, participants responded faster to non-literal words for matching dyads than mismatching dyads. On the other hand, when literal words appeared before images, participants responded faster to matching dyads than mismatching dyads. Non-literal words were rated as more congruous with matching dyads, while literal words were more congruous with mismatching dyads. Thus, non-literal upfix meanings (e.g., inspiration) are ingrained in memory only when they match facial expressions, supporting the notion that they belong to a constrained visual lexicon. Our study contributes a combinatorial method of both verbal and visual modalities into the study of non-literal expressions in memory.

## Introduction

When a lightbulb is depicted above a character’s head, the likely interpretation is that the character is inspired. Elements like these are pervasive in comics, emoji, and across visual media. Yet, lightbulbs do not actually float above people’s heads, and lightbulbs on their own do not evoke “inspiration”. How then do people derive this non-literal meaning by looking at images like these? In this paper we examined this question by an experiment in which images were juxtaposed with elements like lightbulbs next to verbal words that describe the literal (e.g., lightbulb) or non-literal (e.g., inspiration) meanings. This way, we aim to explore the factors that play a role in how people achieve figurative meanings based on these images.

## Theories of visual morphology

These types of above-the-head signs have long been recognized as expressive graphic devices for meaning-making (Forceville, 2011; Kennedy, 1982; McCloud, 1993; Walker, 1980). Previous work has led researchers to conclude that “above-the-head” signs are construed dynamically in context, i.e., “on the fly,” and do call often for metaphoric understanding (Bateman & Wildfeuer, 2014; Forceville, 2011; Kennedy, 1982; McCloud, 1993; Walker, 1980). More recent work, however, had found that while contextual and metaphoric construals play a role in the meaning-making of these elements, the understanding of these signs also relies on conventionalized, stored patterns (Cohn & Foulsham, 2022).

**Fig. 1:**
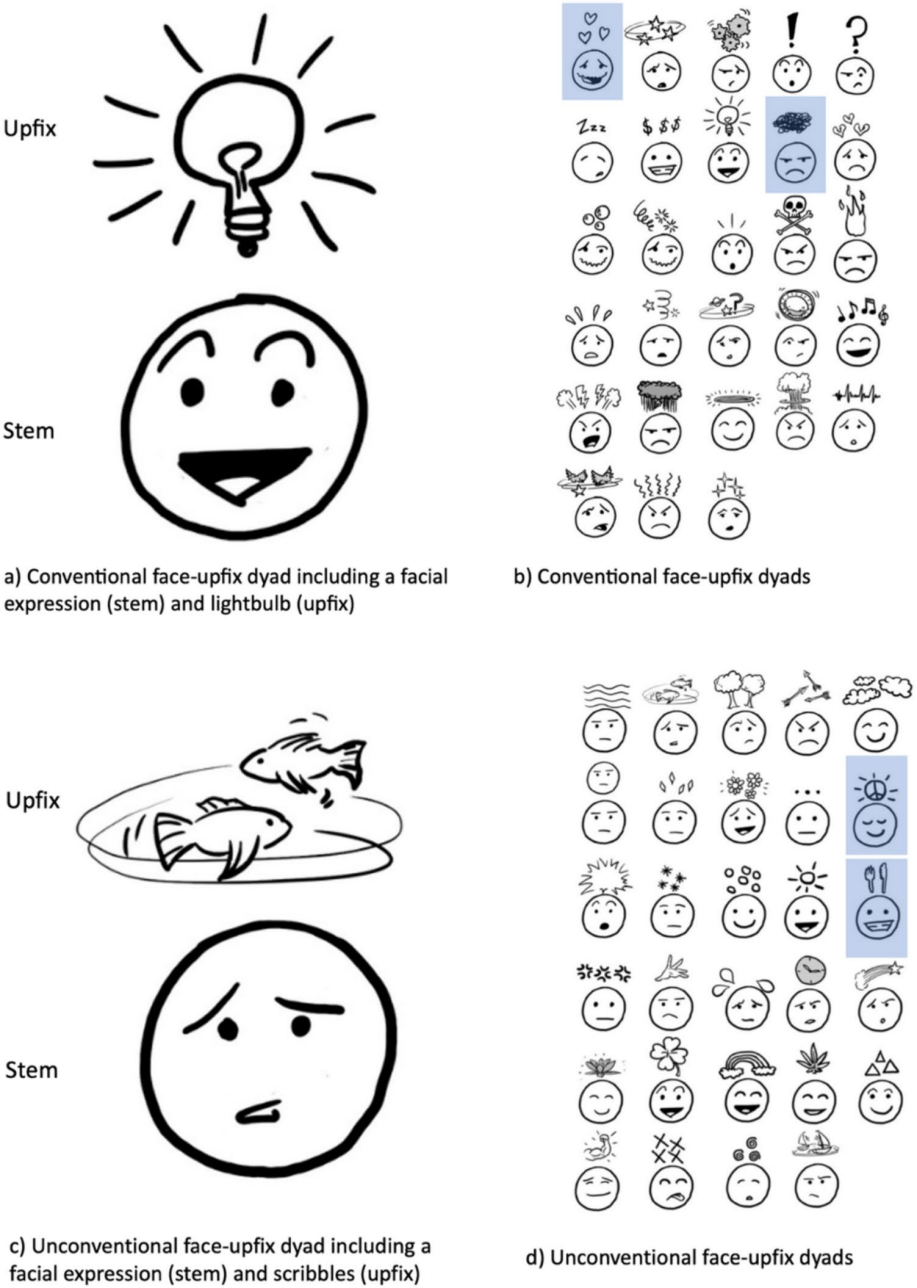
Conventional (**a, b**) and unconventional (**c, d**) matching face-upfix dyads conveying non-literal meanings. Examples of hearts, scribbles, peace sign, and cutlery upfixes are highlighted

This insight aligns with Visual Language Theory (VLT), which proposes that graphics involve comparable (neuro)cognitive structures and processing as language (Cohn, 2013). According to VLT, the lightbulb above the head is an *upfix*, which is a type of “visual lexical item,” a form-meaning mapping stored in long-term memory (Cohn, 2012, 2013), which includes its non-literal meaning. Hence, the lightbulb is just one of several encoded elements that float above the heads of characters, as in Fig. 1, which together suggest that a more abstract schematic template may be at stake.

This idea of an abstracted “lexical schema” for upfixes is consistent with lexical theories of language (Jackendoff & Audring, 2016). Schematic form-meaning mappings can, for example, be identified in the English plural affix -*s* (e.g., one candle vs. multiple candles). This bound morpheme (-*s*) must attach to a stem (e.g., the noun candle), which are often free morphemes that can stand on their own. The plural affix *-s* fulfills a part of a schematic template, containing a slot for the noun and the affix *-s*, that people can apply to various free morphemes in the form of nouns (e.g., plants, cats*,* etc.).

According to VLT, the lightbulb similarly functions as a bound morpheme that attaches to the free morpheme of the face, which can stand alone (Cohn, 2013, 2018; Forceville, 2011). Abstracted across examples, lightbulbs exemplify a kind of visual affix – “upfixes” – that attach to a stem of a character by floating in an upward location relative to their heads (Cohn, Murthy, & Foulsham, 2016). This upfix schema has slots for both the affix (e.g., a lightbulb, or some other element) and a character’s facial expression (see Fig. 1a, c). This abstracted schema manifests in the various conventionalized face-upfix dyads found in comics, emoji, and visual media (Fig. 1a, b) and can give rise to new dyads when novel items are placed above heads (Fig. 1c, d).

The schematic nature of face-upfix dyads is supported by the observation that they obey structural constraints that govern the relationship between the face and the upfix. For example, upfixes need to maintain a spatial position above the face, and upfixes should match the facial expression of the stem character to ensure coherence. Prior work on upfixes has mainly focused on these constraints (Cohn et al., 2016; Cohn & Foulsham, 2022; Han & Choi, 2023; Kendall, Raffaelli, Todd, Kingstone, & Cohn, 2020), but it has not been investigated how they could affect the ability of face upfix dyads to convey non-literal meanings. Therefore, here we investigate which factors contribute to comprehenders’ interpretations of upfixes in a literal or non-literal manner when they see them floating above characters' heads.

**Fig. 2:**
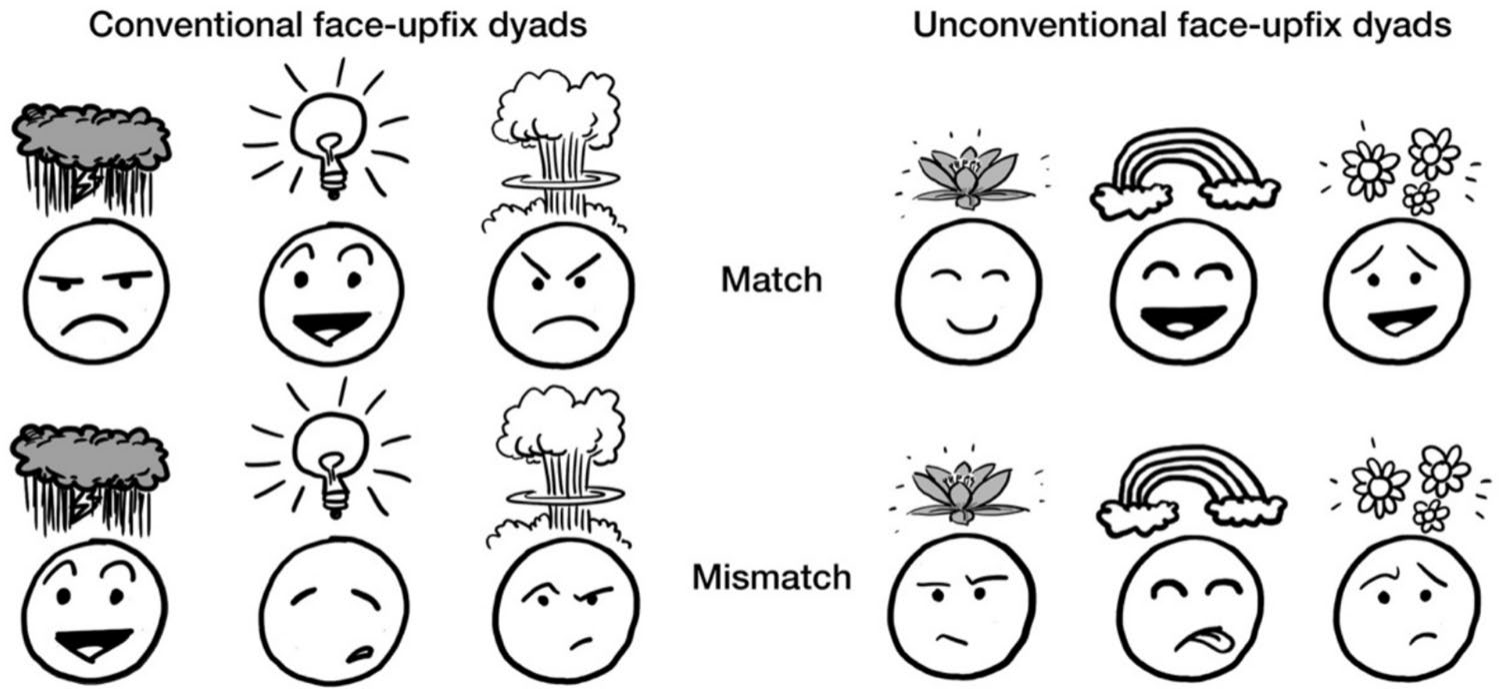
Conventional and unconventional face-upfix dyads where the upfixes and facial expressions are either matching or mismatching

As stated above, one structural constraint that prior work has observed is that upfixes should match the facial expression of their stem character. Given that mismatching dyads contain emotional facial expressions that are inconsistent with the meaning of the upfixes, they violate the coherency of the dyad (Cohn et al., 2016). For example, Fig. 2 shows a storm cloud upfix, which typically refers to a bad mood. This non-literal interpretation matches with an angry face, but mismatches with a happy one. Some upfixes, however, can pair with multiple similar facial expressions (Ojha et al., 2021). For instance, various upfixes related to anger may appear with different angry facial expressions while maintaining coherence. When asked to assess how much faces and upfixes “belong together,” though unconventional dyads are rated as more “mismatching” than conventional dyads, mismatching face-upfix dyads are as worse than matching ones no matter their conventionality. In addition, when participants gave free responses about the meaning of upfix dyads, matching dyads elicited more consistently agreed-upon meanings than mismatching dyads (Cohn et al., 2016). This finding suggests that upfixes need to match with the facial expression of their stem character in order to maintain well-formedness and coherent interpretations of meaning.

However, other emperical studies are divided on whether such matching also manifests in response times. Subsequent research by Kendall et al. (2020) did not find that people responded faster to upfixes matching their stem character compared to upfixes that did not match their stem character when they had to recognize the specific emotions of face-upfix dyads. Response times also did not depend on the style of the faces and/or upfixes (cartoony or photorealitsic). Contrastingly, Cohn and Foulsham (2022) found that people responded faster to matching dyads compared to mismatching dyads while assessing their coherency, and also that, when measured with ERPs, mismatches come with neurocognitive costs to semantic processing, as indiced by greater N400 amplitudes, and subsequent sustained negativities. Unlike Kendall et al. (2020), who compared cartoony with photorealistic images using a limited selection of face-upfix dyads, Cohn and Foulsham's (2022) study included a wide assortment of dyads without any style differences in their stimuli, which might explain the contrasting findings.

This matching constraint appears to be also sensitive to the conventionality of the face-upfix dyad, which refers to how familiar and well ingrained these combinations are in memory. While the effect of mismatching persists for conventional face-upfix dyads, it also affects the interpretation of unconventional dyads (see Fig. 2). That alginment between face and upfix also constraints unconventional dyads supports the idea that people interpret and understand face-upfix dyads using an abstract schema that is stored in their long term memory (LTM), which specifies the general combinatorial structure of such dyads rather than by a concrete set of item-based instances they have been previously exposed to (Cohn et al., 2016).

In addition, while unconventional matching dyads are rated as less congruent than conventional matching dyads, mismatching dyads are consistently assessed as worse than matches no matter the conventionality (Cohn & Foulsham, 2022; Cohn et al., 2016). Furthermore, people responded faster to rate the comprehensibility of matching conventional dyads compared to matching unconventional dyads, but conventiality did not modulate response times to mismatching dyads, which were consistently slower than matching ones (Cohn & Foulsham, 2022). This might be a result of conventional dyads being more ingrained in memory. Moreover, people's comic reading experience, measured with the Visual Language Fluency Index (VLFI), affects this effect of conventionality (Cohn & Foulsham, 2022; Cohn et al., 2016). People more proficient in reading comics had greater differences in their comprehensibility ratings for conventional and unconventional dyads.

Furthermore, conventionality of the face-upfix dyads also manifested in people's neural responses. While costs of semantic processing (N400) were observed for both unconventional dyads and mismatching dyads (though with different scalp distributions), only mismatching dyads evoked sustained negativities, while matching unconvnetional dyads attenuated to be processed similarly to matching conventional dyads (Cohn & Foulsham, 2022). Finally, conventionality modulated brain responses associated with visual pattern recognition (N250), an effect that was also modulated by participants' expertise with comics.

Given this abstract, constrained structure, how might upfixes convey meaning? Within upfixes' consistent structure, they can evoke meaning in relation to their face through several different ways (Cohn, 2018). Some upfixes use fixed signs, which have an encoded meaning independently of their context in upfix dyads (Cohn, 2018; Cohn et al., 2016). For instance, a heart shape is commonly used by people to express the idea of love. When hearts appear as upfixes (as in Fig. 1b), their encoded meaning persists in the face-upfix dyad. This renders the isolated heart symbol and the heart upfix as “sister schemas” (Jackendoff & Audring, 2016), here sharing a form and meaning but differing in morphology, similar to the isolated word “able” and its relation to the affix “-*able*” (as in “construable”). If a peace sign appears as an upfix (as in Fig. 1d), it also usually inherits the fixed meaning of this sign, even though it does not conventionally appear as an upfix. Other signs used as upfixes may have no encoded meaning. These upfixes carry no meaning on their own and cannot be isolated, but create meaning in combination with a character’s facial expressions (Cohn et al., 2016). Consider the dyad with scribbles in Fig. 1b. These scribbles do not convey any fixed independent meaning, but they evoke some state of frustration or anger when combined with the facial expression of the character.

Furthermore, associative links might be created between an upfix and its meaning. For example, an upfix of cutlery (see Fig. 1d), can be associated with hunger due to the semantic association between cutlery and eating. Namely, both cutlerly and hunger are part of the same, more general, semantic field related to food and eating. This commonality, therefore, might cause people to associate the dyad with hunger.

Finally, metaphorical upfixes are characterized by a mapping between two conceptual domains (Forceville, 2011; Kennedy, 1982; Lakoff & Johnson, 1980): the target domain (the concept that is actually talked about) and the source domain (the “added” concept to which the metaphorical meaning applies). The lightbulb upfix (see Fig. 1a) is an example of a metaphorical upfix. Here, a particular state of mind (= the target) is suggested by comparing it to a lightbulb (= the source), such that the mind being in a state of sudden insight or insipiration is similar to a light being turned on. This example instantiates a broader metaphoric frame of *understanding is seeing* (Lakoff & Johnson, 1980), with light reflecting that things can be clearly seen and therefore recognized or perceived. Again, an iconic lightbulb and an upfix lightbulb would be encoded as sister schemas, where they share a form but the different morphology renders them as having different meanings, similar to the relation between “sheep” and “sheepish” which share a form but differ in morphology and semantics (Jackendoff & Audring, 2016).

Across all of these meaning-making strategies, upfixes are able to convey non-literal meanings when combined with facial expressions (Cohn et al., 2016; Ojha et al., 2021). People thus need to combine the meaning of the upfixes with facial expressions to construct non-literal meanings. However, little research has explored the cognitive processes that motivate this kind of combinatory meaning making in a visual context. Therefore, in line with VLT, which predicts common mechanisms persisting across visual and verbal processing, theories about the processing of verbal non-literal meanings could inform some expectations. For example, the word “blue” (e.g., as a color) does not evoke a non-literal meaning (e.g., the concept of “feeling sad”) on its own, but it can emerge when combined with other words in a sentence (e.g., “I am feeling blue”).

## Theories of verbal non-literal meaning-making

One theory that explains how we can interpret this sentence as “I am feeling sad” is proposed by Grice (in de Grauwe et al., 2010) and posits a *serial processing view*. To process the intended non-literal meaning of sentences like “the dress fits like a glove,” people are thought to first compute an ill-formed literal meaning (e.g., the dress fits like a cover for the hand that protects one from dirt) before construing a non-literal meaning (e.g., the dress is the right size and well suited). According to this theory, processing figurative and literal sentences qualitatively differ from each other. In line with this view, people would first literally interpret the dyad in Fig. 1 as “an actual lightbulb flying above a character’s head” before computing a non-literal meaning including that “this character is inspired.”

A second theory, contrasting with the first one, is called the *direct access model* (Gibbs, 2002). This theory proposes that people can access the non-literal meaning of a sentence directly, without first computing a literal meaning, so long as the sentence context supports its non-literal meaning. In this model, context plays a key role in deciding which meaning will be accessed initially. According to this view, people would literally interpret the dyad in Fig. 1 if it is placed in a context that supports this interpretation (e.g., a story world in which it is normal that actual objects fly above characters' heads). On the other hand, when the context supports its non-literal meaning (e.g., before the lightbulb appeared above the characters head, it was trying to solve a problem), people can directly interpret the character in Fig. 1a as “being inspired.”

A third theory is called the *graded salience model* by Giora (1997). According to this theory, both literal and non-literal meanings are available to interact with context and the semantic “salience” of a word helps determine its processing. When the non-literal meaning of a word is conventionalized (e.g., “stone” conventionally expresses “unkindness” or “cruelness” in sentences like “she has a heart of stone”), it will initially be accessed because it seems to be more salient than the literal meaning (e.g., her heart consists of stone as a material). This activation will even occur when the non-literal meaning is not reinforced by the context in idioms. Infrequent, unconventional non-literal meanings will be less salient, leading to the literal meaning being accessed first (even when metaphorical meaning is reinforced by the context). The estimated relevance of the activated meaning will determine whether it will be retained or suppressed at an ulterior stage of processing. In line with this, conventional dyads (Fig. 1a, b) will be more likely to be directly interpreted in a non-literal manner, compared to unconventional dyads (Fig. 1c, d).

The above-mentioned models propose ideas about when and how people construct non-literal meanings out of verbal information like sentences, and could also potentially explain how people derive non-literal meanings from visual information in the form of face-upfix dyads. A frequently used method to investigate meaning-making processes across different modalities is cross-modal priming, a paradigm in which a stimulus in one modality (e.g., a written word) is shown before a stimulus in another modality (e.g., an image). Prior work on the differences between words and picture priming found that people responded faster to words followed by pictures than pictures followed by words when they are asked to perform a same-different categorical judgment task (Pellegrino et al., 1977). This effect was also found for words and pictures that had a congruent relationship (Van Der Meer et al., 2003). Indeed, a recent study on emoji showed that participants responded faster to emoji that were congruent with a preceding word compared to those that were incongruent when they had to perform a match/mismatch task, and that this effect was modulated by the recognized conventionality of the emoji meanings (Weissman et al., 2023).

Another study by Valenzuela and Soriano (2007) showed that response times are sensitive to the relatedness of picture-word primes. In this study, participants had to perform a semantic categorization task of a word after being primed with a related or unrelated picture across four different categories (tools, vehicles, fruit, and anger). In addition, they found that people made faster decisions if related prime pictures were followed by concrete words (picture of “pliers” followed by the word “hammer”) than abstract words (picture of “lava” followed by the word “wrath”).

While this may imply that literal meanings, being more concrete, would be easier to process than non-literal meanings, as abstractions, this is not always the case. Cacciari and Tabossi (1988) showed that when people were primed with predictable idiomatically related sentences (familiar idioms such as “The tennis player was in seventh heaven”), they responded faster to idiomatically related words (e.g., “happy”) compared to literal words (e.g., “stars”), when they had to perform a lexical decision task. In contrast, the reverse was found when people were primed with unpredictable idiomatic sentences (unfamiliar idioms). Moreover, Titone and Connine (1994) showed that literal meanings of highly predictable idioms were only activated simultaneously with their idiomatic meanings if their literal interpretations were highly plausible when people performed a lexical decision task. For highly predictable idioms that lacked a plausible literal interpretation, only idiomatic meanings were activated.

Given these precedents, we used cross-modal priming to examine which factors contribute to people constructing literal or non-literal meanings when they view face-upfix dyads. This study builds on previous work that mainly focused on upfixes and their structural constraints in the visual modality (Cohn & Foulsham, 2022; Cohn et al., 2016; Han & Choi, 2023; Kendall et al., 2020; Ojha et al., 2021). Furthermore, this study explores whether combinatory meaning making in a visual context exhibits cognitive processes similar to verbal combinatory meaning making. To do so, we showed participants face-upfix dyads before or after words which either stressed the upfix’s literal meaning (e.g., lightbulb) or its non-literal meaning (e.g., inspiration). The dyads were also manipulated to either have matching face-upfix relations or mismatching relations (as in Fig. 2). Participants were asked to judge the congruity between these words and dyads, while we measured both their responses and the time it took to respond.

## Predictions

Based on prior findings from cross-modal priming that indicated that people respond faster to concrete than abstract words that were primed with related pictures (Valenzuela & Soriano, 2007), we first predicted that, in general, literal words associated with face-upfix dyads will be responded to faster and be perceived as more congruous than non-literal words. In addition, participants were expected to respond faster to matching dyads and perceive them as more congruous compared to mismatching dyads, as was found in prior work (Cohn & Foulsham, 2022; Cohn et al., 2016). Finally, we posited that participants would respond faster to dyads presented before words than to dyads presented after words, benefiting from the facilitation of the preceding images (Pellegrino et al., 1977; Van Der Meer et al., 2003).

We also considered interactions between word type (literal, non-literal), matching (match, mismatch) and order (word-image, image-word). For mismatching dyads, we expected participants to respond faster and give higher congruity judgments to literal meanings compared to non-literal meanings. Namely, literal meanings (e.g., lightbulb) are represented solely in the upfixes, through which they can be identified in both matching and mismatching dyads. In contrast, non-literal meanings (e.g., inspiration) require the compositional meaning of upfixes and faces, which is unavailable in mismatching dyads (Cohn et al., 2016). For matching dyads, we predicted the opposite, with non-literal meanings responded to faster and perceived as more congruous than literal meanings, given that these could be ingrained in memory, which may decrease the plausibility of literal interpretations (Cacciari & Tabossi, 1988; Cohn et al., 2016; Titone & Connine, 1994). We also explored whether the order in which the words and images were presented, affected this interaction.

Finally, earlier studies have found that people’s proficiency in reading comics has affected the magnitude of difference in interpretations of conventional and unconventional face-upfix dyads. To control for this possibility, we decided to include the conventionality of the face-upfix dyads and participants comic reading experience.

## Methods

### Stimuli

We used 56 pre-existing face-upfix dyads that varied in their ratings of conventionality, with rating scores for all stimuli obtained by Cohn et al. (2016). These ratings were based on the degree of agreement across participants' free responses when interpreting face-upfix dyads. This measure for conventionality has demonstrated reliability across several studies for capturing this variance (Cohn & Foulsham, 2022; Cohn et al., 2016). The 56 matching upfixes (i.e., the facial expression matched with the meaning of the upfix) were paired with 56 mismatching upfixes (i.e., the facial expression and meaning of the upfix did not match with each other), which also had ratings of conventionality. Each dyad was paired with two words: 56 literal, 56 non-literal. Participants either viewed words before images or images before words. This rendered each scenario with eight conditions that manipulated factors of matching (match, mismatch), word type (literal, non-literal) and stimuli order (word-image, image-word).

As each participant was exposed to all four matching and word type conditions, the words and face-upfix dyads were distributed into four lists of 56 trials that were counterbalanced in a Latin square design so that participants viewed each upfix and word only once and had 14 trials of each condition, presented in a randomized order or each participant. Each list also included 15 filler trials that varied in terms of word type and matching. The visual signs in the filler trials had a different placement relative to the face (e.g., placed on the character’s forehead, eyes, nose, ears or mouth).

**Fig. 3:**
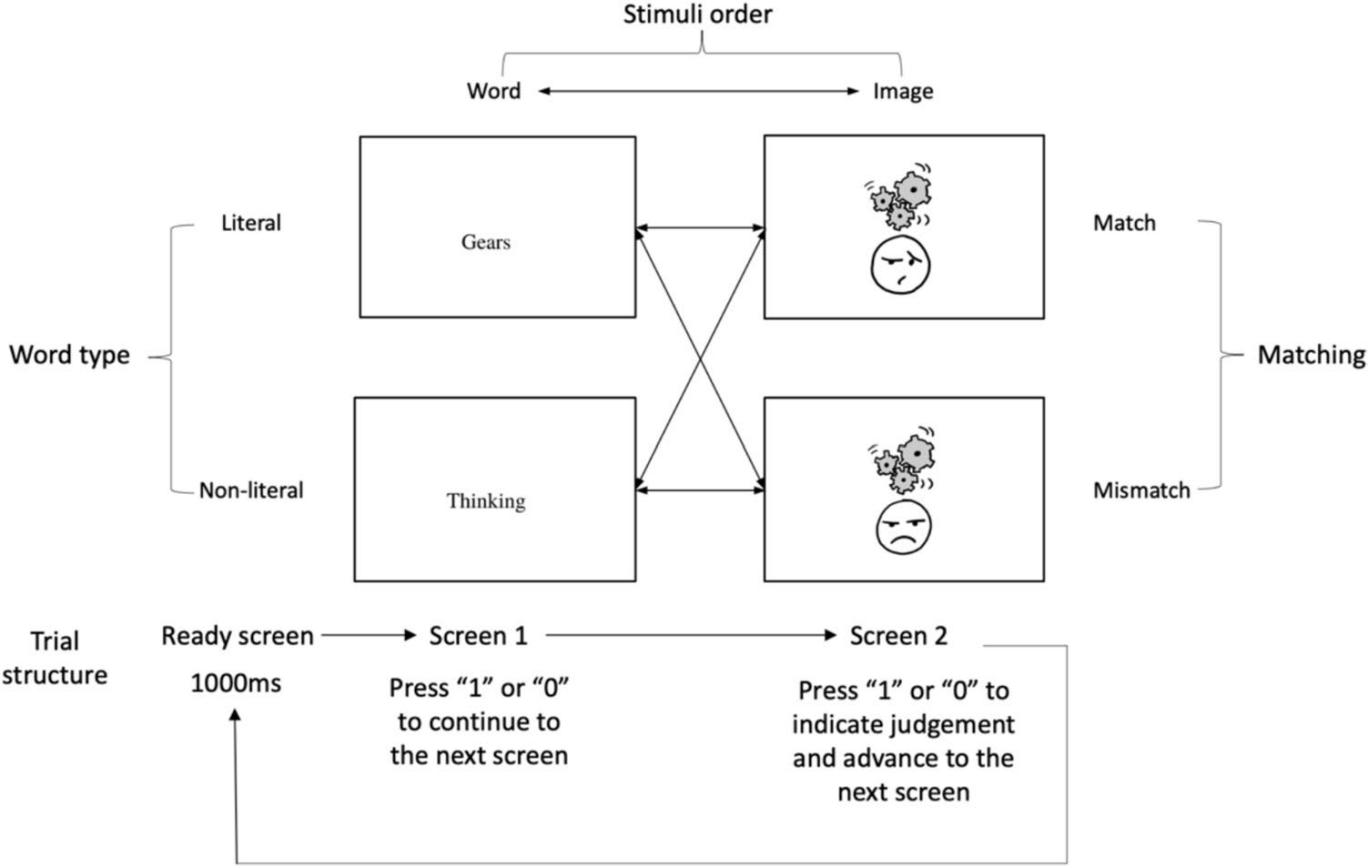
Overview of all trials across all matching, word type and stimuli order conditions, combined with the trial structure

Each trial consisted of both a word and an image. To prevent that we could ascribe certain findings to the order in which we used the primes, the order in which words and images were presented was different for two groups of participants. In one group, the words were presented before the images, while participants in the other group viewed the images before the words. Thus, while matching and word type were within-subjects factors, order was a between-subjects factor. Example scenarios of all conditions can be seen in Fig. 3.

### Participants

One hundred and twenty-two students from Tilburg University (43 males, 79 females, mean age: 20.3 years, *SD* = 3.3 years), completed the experiment via an online survey. Given that one participant performed outside the outlier range (see below), the data of only 121 participants was analyzed. Before the experiment started, participants were asked to fill out the Visual Language Fluency Index (VLFI), which is a questionnaire that assesses participants'visual language expertise via questions about how often they had read several types of visual narratives (e.g., graphic novels, comic strips, comic books, etc.). These were measured on a scale of 1 to 7 (1 = never, 7 = always). The questionnaire also addressed how often they had drawn comics (both during their childhood and now), which was measured on a five-point scale (1 = below average, 5 = above average). VLFI scores have been widely shown to be a reliable measure of expertise to capture the variance in both behavioral and neurocognitive methods (Cohn, 2020). In general, low fluency is indicated by VLFI scores below 8, average around 12, and high around 22. The mean VLFI score of the participants in this experiment can therefore be considered as low (*M* = 8.79, *SD* = 5.79, range: 1.5–29).

### Procedure

Participants used a web browser to complete the online experiment via Qualtrics using the lab.js JavaScript plugin (Henninger et al., 2021). All the participants gave their informed written consent, which was obtained through a confirmatory button press. Prior to experimentation, participants filled out the VLFI. When the experiment began, they were presented with 56 trials that consisted of both a word and an image following a 1,000-ms “Ready” screen.

The specific trial structure differed across two groups, but all screens were self-paced with no fixed durations (see Fig. 3). In one group, the first screen of each trial consisted of either a literal or non-literal word and an informing text that noted that participants could press “1” or “0” to continue to the next screen. After the participants pressed their choice, the experiment continued to the second screen. In the second screen, participants were presented with a face-upfix pair, which was either matching or mismatching. Beneath the image, participants were asked to judge the congruity between the face-upfix dyad and the word on the previous screen (1 = yes, 0 = no) and they advanced to the next screen by pressing their choice. In the other group, the stimuli were presented in reversed order: participants viewed face-upfix dyads on the first screen and were presented with words on the second screen. Participants were assigned randomly to the different groups and trials within each group were presented in a randomized order. After finishing the experiment, participants filled out a short open-response post-test questionnaire, in which they could report possible observed patterns or things that they perceived as “unusual” throughout the experiment. The whole experiment took around 30 min to complete.

### Data analysis

To analyze the effect of matching, word type and stimuli order on response times and congruity judgments, two repeated measures mixed model ANOVAs were used. For response times, we removed outliers that were below 300 ms or above 10,000 ms. Both ANOVAs contained two within subject factors (matching: match vs. mismatch; word type: literal vs. non-literal) and one between subject factor across groups (stimuli order: word-image vs. image-word). Follow-up post hoc comparisons used Bonferroni corrections. We considered that congruity judgments (1 “yes,” and 0 “no”) were taken to reflect participants' personal intuitions, which is why we did not classify their accuracy as “correct” or “incorrect”.

In addition, to test the influence of visual language expertise and the conventionality score of the face-upfix dyads, analyses were performed that included the VLFI scores and conventionality scores as covariates in the two repeated measures mixed model ANOVAs. Lastly, correlation analyses followed up significant effects to explore relations between conventionality and response time and congruity judgments.

We chose to treat the binary congruity judgments as a subject measure. This is because we wanted to determine the degree to which participants internally agreed about the congruity between faces and upfixes across matching, word type and stimuli order conditions. Therefore, we calculated mean judgment scores for each participant per condition (used in the repeated-measures ANOVA and repeated-measures ANCOVA for visual language expertise). Furthermore, to assess the degree to which participants' judgments differed between each other, we also calculated mean judgment scores for each possible combination of words and images across stimuli order conditions (used in the repeated-measures ANCOVA for conventionality).

## Results

### Response time

**Fig. 4:**
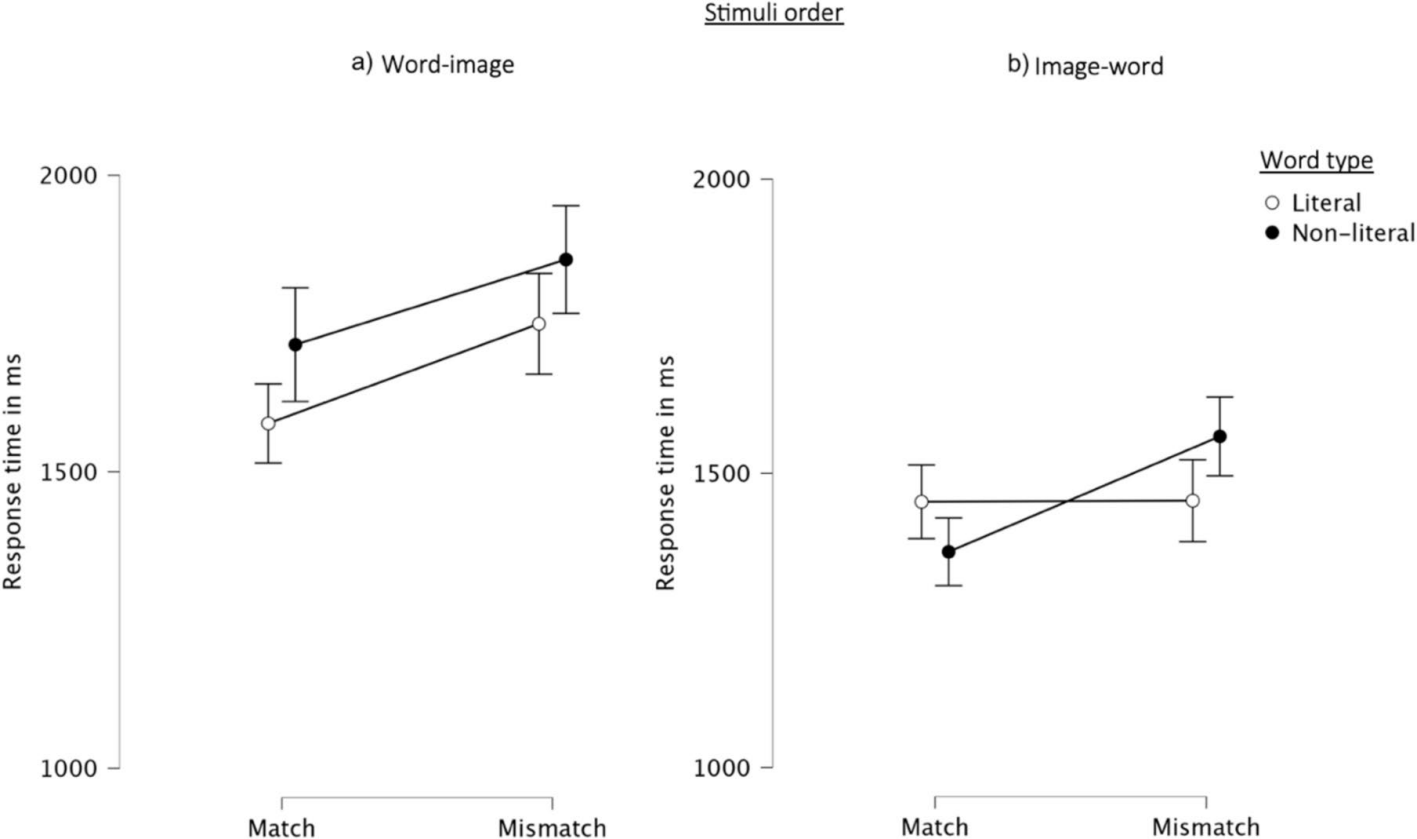
Response times (ms) given (**a**) to images of matching and mismatching dyads preceded by literal and non-literal words and (**b**) literal and non-literal words preceded by images of matching and mismatching dyads. Error bars display 95% confidence intervals

The analysis of response times^1^ revealed main effects for word type, matching and stimuli order, and an interaction between them (see Table 1). As depicted in Fig. 4ab, participants, generally, responded faster to judge the congruity of word-upfix relationships with literal than non-literal words. In addition, matching dyads led to faster response times than mismatching ones. Lastly, participants who responded to words preceded by images (image-word order) responded faster than participants who responded to images preceded by words (word-image order).

**Table 1:** Results of repeated-measures mixed ANOVAs, comparing the effect of matching, word type and stimuli order for response time and congruity judgments

	Response time		Congruity judgments	
	F-value	η_p_^2^	F-value	η_p_^2^
Word type (WT)	5.01*	0.04	33.79***	0.22
Matching (M)	25.10***	0.17	234.45***	0.66
Stimuli order (SO)	14.81***	0.11	0.42	0.00
WT * M	2.96^	0.02	158.58***	0.57
WT * SO	3.40^	0.03	0.25	0.00
M * SO	1.24	0.01	0.66	0.01
M * WT * SO	4.91*	0.04	0.57	0.01

df = 1,120.

***p < 0.001.

**p < 0.01.

*p < 0.05.

^*p* < 0.1

In further analyzing the interaction between word type, matching and stimuli order, post hoc tests revealed that in almost all conditions people were faster to judge congruity when they responded to words preceded by images compared to when they responded to images preceded by words. In the mismatch conditions with literal words, participants responded faster to words preceded by images than to images preceded by words (*Mdif* = −296.13, *p* = 0.013, CI [32.74; 559.52]). Across both matching (*Mdif* = −347.89, *p* = 0.001, CI [84.50; 611.28]) and mismatching (*Mdif* = −295.23, *p* = 0.013, CI [31.84; 558,62]) conditions with non-literal words, people also responded faster to words preceded by images than images preceded by words. This effect was, however, not found for conditions including both matching dyads and literal words. The order in which they were presented did not affect participants’ response times to judge the congruity of these word-image pairs.

We also found different effects of word type across matching and stimuli order combinations. Participants responded faster to images of matching dyads than mismatching dyads preceded by literal words (*Mdif* = −167.99, *p* = 0.03, 95% CI [−327.57; −8.42]), see Fig. 4a. On the other hand, participants responded faster to non-literal words preceded by matching dyads compared to mismatching dyads (*Mdif* = −196.15, *p* = 0.003, CI [−353.12; −39.17]), see Fig. 4b.

**Fig. 5:**
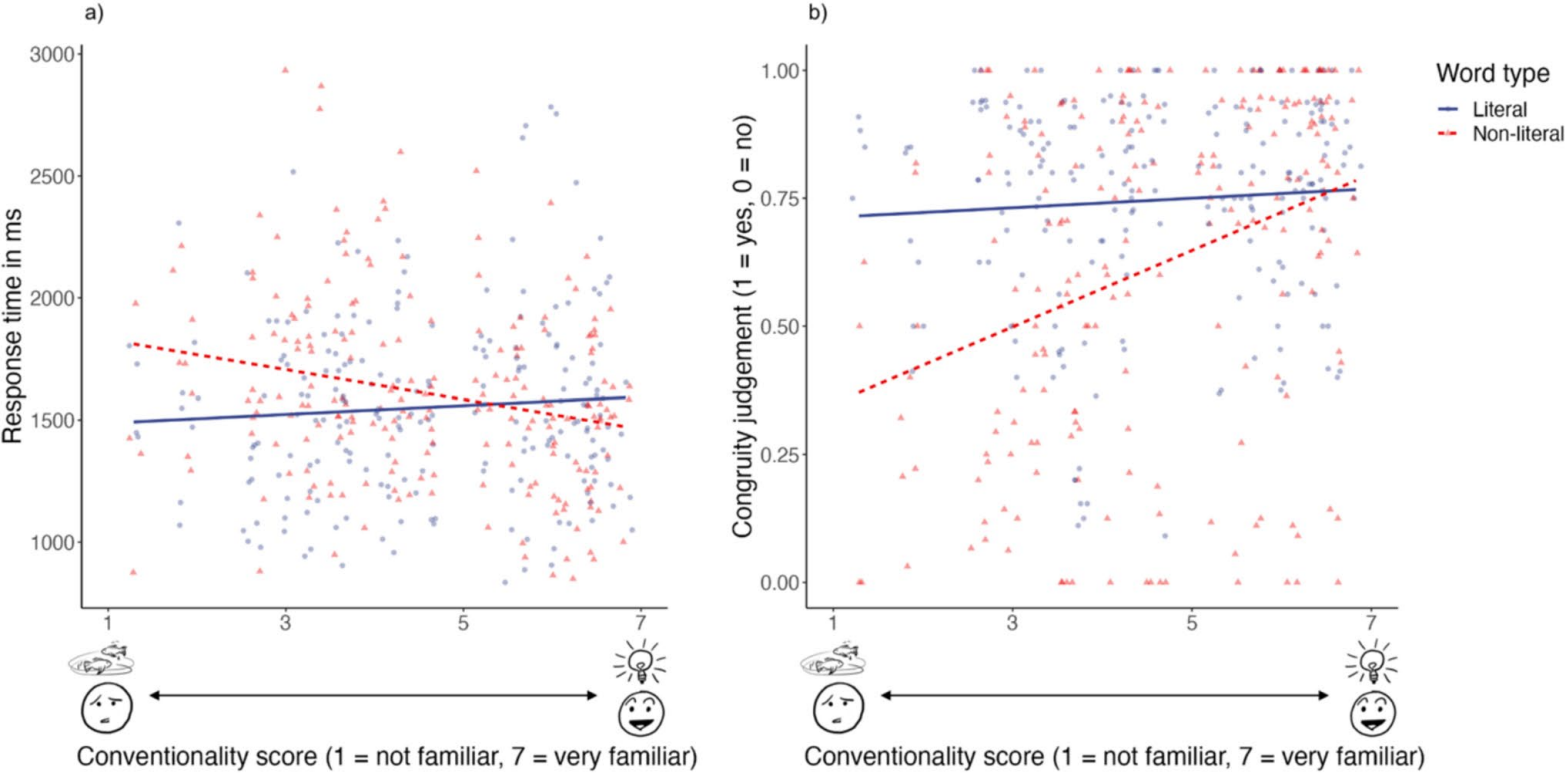
(**a**) Mean response times in ms and (**b**) congruity judgments (1 = yes, 0 = no) for participants to face-upfix dyads that differed in terms of conventionality scores (1 = not familiar, 7 = very familiar) for both literal and non-literal words

Conventionality also interacted with word type, *F*(1,103) = 6.82, *p* = 0.01, partial *η*^*2*^ = 0.06. As shown in Fig. 5a, while greater conventionality of upfixes led to faster response times for dyads with non-literal words, *r*(211) = −0.22,* p* = 0.001, no such relationship was observed for literal words, *r*(211) = 0.04,* p* = 0.60. No significant effects were found for analyses that included VLFI scores.

### Congruity judgments

**Fig. 6:**
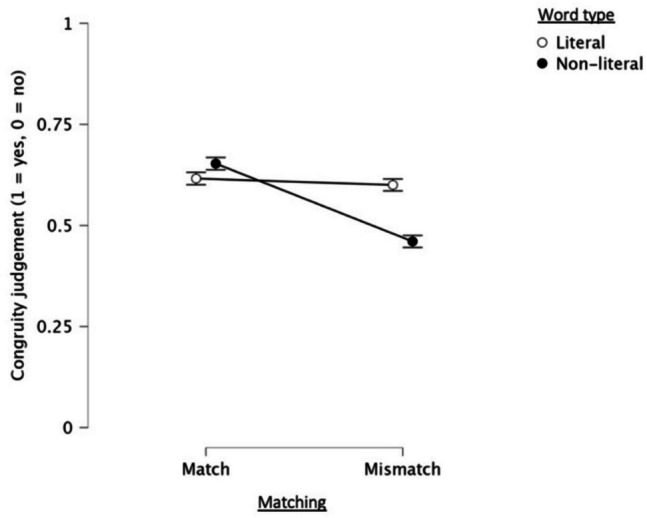
Mean congruity judgments (1 = yes, 0 = no) given to literal and non-literal words for both matching and mismatching face-upfix dyads. Error bars display 95% confidence intervals

We next analyzed judgments of how well the words and face-upfix dyads matched each other (1 = yes, 0 = no). Main effects appeared for word type and matching, along with an interaction between them (see Table 1), but no main effects or interactions appeared with order. As can be seen in Fig. 6, participants judged combinations of literal meanings and dyads to be more congruent compared to combinations of non-literal meanings and dyads. Furthermore, matching dyads were also judged to be more congruent than mismatching dyads.

Post hoc tests were run to analyze the interaction between word type and matching. Irrespective of their presentation order, participants judged combinations of non-literal words and matching dyads to be more congruent than combinations of literal words and matching dyads (*Mdif* = 0.04, *p* = 0.008, CI [−0.07; −0.01]). Combinations of non-literal words and matching dyads were also judged to be more congruent than combinations of non-literal words and mismatching dyads (*Mdif* = 0.19, *p* < 0.001, CI [0.17; 0.22]). Yet, combinations of literal words and mismatching dyads were perceived as more congruous than combinations of non-literal words and mismatching dyads (*Mdif* = 0.14, *p* < 0.001, CI [0.11; 0.17]).

In addition, conventionality scores interacted with word type, *F*(1,103) = 5.41, *p* = 0.02, partial *η*^*2*^ = 0.05. As depicted in Fig. 5b, greater conventionality of upfixes led to higher mean congruity judgments for both literal (*r*(211) = 0.16, *p* = 0.02) and non-literal words (*r*(211) = 0.30, *p* < 0.001), regardless the presentation order of the stimuli. This suggested that dyads with high conventionality scores were perceived as more congruous with all words than dyads with low conventionality scores. No significant effects could be found in analyses that included VLFI scores.

## Discussion

This study investigated how people construe literal or non-literal meanings during visual combinatory semantic processing in face-upfix dyads. Previous studies comparing literal and non-literal meanings mostly focused on combinatory semantic processing in a spoken language context (see de Grauwe et al., 2010; Gibbs, 2002; Giora, 1997), while studies of face-upfix dyads primarily focused on their structural constraints in a visual language context (Cohn & Foulsham, 2022; Cohn et al., 2016; Kendall et al., 2020). Therefore, we chose to combine both the verbal and visual modality in a cross-modal priming experiment. We measured participants' response times and congruity judgments to either a literal or non-literal word before either a matching or mismatching face-upfix dyad (or the other way around).

Across all stimuli and presentation orders, participants responded faster to judge word-image congruity for literal than non-literal words. This aligns with previous cross-modal priming research showing that literal words are easier to process than non-literal words (Valenzuele & Soranjo, 2007). Literal words were, generally, also judged to be more congruent with face-upfix dyads compared to non-literal words, irrespective of the order in which they were presented. While the order of the stimuli may have affected how participants interacted with the verbal prompt (i.e., being primed by it when the word preceded an image or trying to recognize its meaning in a visual when an image preceded the word), in both cases literal meanings (e.g., lightbulb) have the advantage over non-literal meanings to be recognizable across all face-upfix dyads because their meanings are solely presented in the upfix. In contrast, non-literal meanings (e.g., inspiration) require people to combine face and upfix meanings, through which they can only be recognized in matching and not in mismatching dyads (Cohn et al., 2016).

The structural constraint of matching was also found to affect people's response times and congruity judgments. Across all stimuli, participants were faster to judge the congruity between words and matching dyads than between words and mismatching dyads, aligning with prior work in which people responded faster to matching dyads than mismatching dyads while assessing their coherency (Cohn & Foulsham, 2022). In addition, matching dyads were judged to be more congruent with the presented words compared to mismatching dyads. This is in line with previous research in which matching dyads led to more consistent and higher ratings for familiarity and comprehensibility compared to mismatching dyads (Cohn et al., 2016).

Furthermore, the effect of matching was different for literal and non-literal words. When participants judged the congruity between dyads and literal words (regardless the order in which they were presented), it did not matter whether upfixes matched facial expressions. Again, this suggests that literal meanings can be identified regardless of matching because they are visualized by the upfix alone. On the other hand, matching did affect people’s congruity judgments about dyads and non-literal words. Non-literal meanings were judged to be a better fit to matching dyads than to mismatching dyads, which supports that they require the combination of face and upfix meanings (Cohn et al., 2016). Moreover, matching dyads were judged to be more congruent with non-literal than literal meanings, while mismatching dyads were perceived to be a better fit with literal than non-literal meanings, irrespective of the presentation order. This aligns with the idea that non-literal meanings of matching dyads are possibly ingrained in memory, which may make it less likely that people interpret matching dyads in a literal manner (Cacciari & Tabossi, 1988; Cohn et al., 2016; Titone & Connine, 1994). As this interaction only affected participants' congruity judgments and not their response times, the results only partially confirm our predictions. The effect of stimuli order is dicussed below.

The order of words and images also influenced the response times to dyads in combination with their literal or non-literal meanings. In line with prior work (Pellegrino et al., 1977; Van Der Meer et al., 2003), participants responded faster to judge words preceded by images than to images preceded by words. This effect of stimuli order was, however, different for matching dyads presented with literal words. Participants’ response times for this combination did not differ, regardless the order in which they were presented. These findings support that, on the one hand, the time to activate literal meanings of matching dyads is not different for word and image priming. On the other hand, the results suggest non-literal meanings are easier to retrieve after image priming compared to word priming.

We also found that stimuli orders also varied based on the influence of matching on the congruity between dyads and literal or non-literal meanings. Where matching affected participants’ response times to judge non-literal words after image priming, it did not affect response times to images preceded by words with literal meanings. This supports that matching dyads can more directly activate non-literal meanings than mismatching dyads, since matching dyads are ingrained in memory (Cohn et al., 2016), leading to easier access of the non-literal meaning of the dyad and resulting in faster responses (Cacciari & Tabossi, 1988; Titone & Connine, 1994).

Moreover, matching dyads appeared to require less effortful processing compared to mismatching dyads when presented after literal words. This difference supports that matching dyads are easier to comprehend and more familiar than mismatching dyads (Cohn & Foulsham, 2022; Cohn et al., 2016). However, since they are cued only by the upfix, literal meanings can be derived from both matching and mismatching dyads. Therefore, the order of the stimuli only affected participants' processing time and not their conscious intuitions about congruity.

Returning to the verbal theories outlined in the introduction, these findings contrast the expectations of the serial processing view where literal meanings are always computed before non-literal meanings (Grice, in de Grauwe et al., 2010). We found no differences in response times between literal and non-literal meanings when they were presented before or after matching dyads. Participants also perceived matching dyads as a better fit for non-literal than literal words, regardless the order in which they were presented. This indicates that for matching dyads, non-literal meanings are more salient than literal meanings. Instead, these findings support Giora's (1997) graded salience model where the most salient meaning (which can both be literal and non-literal) is activated during non-literal combinatory semantic processing.

In addition, the graded salience model is further supported by the variance across matching face-upfix dyads based on their conventionality (Cohn & Foulsham, 2022; Cohn et al., 2016). More conventional dyads that were presented before or after non-literal words led to faster response times and higher congruity ratings than less conventional dyads, similar to results observed to word-emoji matching which also varied based on their conventionality (Weissman et al., 2023). This suggests that non-literal meanings of conventional face-upfix dyads are more entrenched in people's memory, as they are more common, compared to unconventional ones. This finding also contrasts an interpretation of direct access processing (Gibbs, 2002), since it is not solely the context, but also the salience of the meaning determining which will be accessed first.

We did, however, not find any effects regarding participants’ comic reading experince. This contrasts prior work in which comic reading proficiency modulated participants' processing of dyads that ware manipulated in terms of structural properties such as matching and conventionality (Cohn & Foulsham, 2022; Cohn et al., 2016). The lack of such findings in this study could potentially be attributed to the overall low comic reading experience of the participants. As a result of this unbalanced sample, the study might lack statistical power to detect any effects regarding comic reading proficiency. Future research is, therefore, encouraged to include a sample that is equally balanced in terms of low and high experienced participants.

## Conclusion

Altogether, these findings suggest that the salience of the meaning is an important factor in determining whether people access literal or non-literal meanings during visual combinatory meaning-making (Giora, 1997). Given that a model about verbal combinatory meaning making can be applied to visual combinatory meaning making, it supports the applicability of theories of non-literal meanings across modalities.

Finally, these results also further support that non-literal meanings for matching face-upfix dyads (e.g., inspiration) are ingrained in memory, belonging to a constrained visual lexicon (Cohn, 2012, 2013). People can process new, unconventional dyads that lack a salient non-literal meaning, but regardless their novelty, the processing of these dyads still can be violated by the matching constraint. This supports the idea that people use an abstract schematic template including slots for both upfixes and facial expressions during the processing of face-upfix dyads. As this is consistent with lexical theories of language (Jackendoff & Audring, 2016), such findings again suggest alignment in structure and processing of the verbal and visual modalities, supporting that common mechanisms persist across multiple modalities.

## Data Availability

The data are publicly available via https://doi.org/10.34894/RMVZJV

## Footnotes

1 We performed a one-way ANOVA to check whether there was an effect of response (yes vs. no) on response times. We found that people, in general, were faster to give a “no” response, compared to a “yes” response (*F*(1,16,642) = 57,869, *p* <.001, partial *η*^*2*^ = 0.003). Given that there was an almost equal distribution of the number of “yes” (*N* = 8054) and “no” (*N* = 8590) responses, possible effects can be canceled out.

## Declarations

### Open Practices Statement

The materials used for the experiment are available upon request of the authors. The experiment was not preregistered.

## References

[CR1] Bateman, J. A., & Wildfeuer, J. (2014). Defining units of analysis for the systematic analysis of comics: A discourse-based approach. *Studies in Comics,**5*(2), 373–403.

[CR2] Cacciari, C., & Tabossi, P. (1988). The comprehension of idioms. *Journal of Memory and Language,**27*(6), 668–683.

[CR3] Cohn, N. (2012). Explaining “I can“t draw“: Parallels between the structure and development of language and drawing. *Human Development,**55*(4), 167–192.

[CR4] Cohn, N. (2013). *The visual language of comics: Introduction to the structure and cognition of sequential images*. Bloomsbury.

[CR5] Cohn, N. (2018). Combinatorial Morphology in Visual Languages. In *Combinatorial morphology in visual languages* (Vol. 4, pp. 175–199 (CHAPTER 7)).

[CR6] Cohn, N. (2020). Visual narrative comprehension: Universal or not? *Psychonomic Bulletin & Review,**27*(2), 266–285. 31820277 10.3758/s13423-019-01670-1 PMC7093370

[CR7] Cohn, N., & Foulsham, T. (2022). *Meaning above (and in) the head: Combinatorial visual morphology from comics and emoji*. *50*(7), 1381–1398. 10.3758/s13421-022-01294-2 PMC9508049 35235175

[CR8] Cohn, N., Murthy, B., & Foulsham, T. (2016). Meaning above the head: Combinatorial constraints on the visual vocabulary of comics. *Journal of Cognitive Psychology,**28*(5), 559–574.

[CR9] de Grauwe, S., Swain, A., Holocomb, P. J., Ditman, T., & Kuperberg, G. R. (2010). Electrophysiological insights into the processing of nominal metaphor. *Neuropsychologia,**48*(7), 1965–1984. 20307557 10.1016/j.neuropsychologia.2010.03.017 PMC2907657

[CR10] Forceville, C. (2011). Pictorial runes in Tintin and the Picaros. *Journal of Pragmatics,**43*(3), 875–890.

[CR11] Gibbs, R. W. (2002). A new look at literal meaning in understanding what is said and implicated. *Journal of Pragmatics,**34*(4), 457–486.

[CR12] Giora, R. (1997). Understanding figurative and literal language: The graded salience hypothesis. *Cognitive Linguistics,**8*(3), 183–206.

[CR13] Han, H., & Choi, J. (2023). Emotion Recognition in Comics: The Effect of Visual Morphemes in Visual Narrative Contexts. *Journal of Cognitive Science*, *24*(3).

[CR14] Henninger, F., Shevchenko, Y., Mertens, U. K., Kieslich, P. J., & Hilbig, B. E. (2021). lab. js: A free, open, online study builder. *Behavior Research Methods*, 1–18. 10.3758/s13428-019-01283-5 PMC9046347 34322854

[CR15] Jackendoff, R., & Audring, J. (2016). *Morphological Schemas. the Mental Lexicon,**11*(3), 467–493.

[CR16] Kendall, L. N., Raffaelli, Q., Todd, R. M., Kingstone, A., spsampsps Cohn, N. (2020). Show me how you feel: Iconicity and systematicity in visual morphology. *In P. Perniss, O. Fischer, spsampsps C. Ljungberg (Eds.), Operationalizing iconicity (iconicity in language and literature series)*, *11*, 214–229.

[CR17] Kennedy, J. M. (1982). Metaphor in pictures. *Perception,**11*(5), 589–605. 6193482 10.1068/p110589

[CR18] Lakoff, G., & Johnson, M. (1980). Conceptual metaphor in everyday language. *The Journal of Philosophy,**77*(8), 453–486.

[CR19] McCloud, S. (1993). Understanding comics: The invisible art. *Northampton, Mass,**7*, 4.

[CR20] Ojha, A., Forceville, C., & Indurkhya, B. (2021). An experimental study on the effect of emotion lines in comics. *Semiotica,**2021*(243), 305–324.

[CR21] Pellegrino, J. W., Rosinski, R. R., Chiesi, H. L., & Siegel, A. (1977). Picture-word differences in decision latency: An analysis of single and dual memory models. *Memory & Cognition,**5*(4), 383–396. 24203005 10.3758/BF03197377

[CR22] Titone, D. A., & Connine, C. M. (1994). Comprehension of idiomatic expressions: Effects of predictability and literality. *Journal of Experimental Psychology: Learning, Memory, and Cognition,**20*(5), 1126. 7931098 10.1037//0278-7393.20.5.1126

[CR23] Valenzuela, J., & Soriano, C. (2007). Looking at metaphors: A picture-word priming task as a test for the existence of conceptual metaphor. *Barcelona English Language and Literature Studies,**16*, 1–15.

[CR24] Van Der Meer, E., Friedrich, M., Nuthmann, A., Stelzel, C., & Kuchinke, L. (2003). Picture–word matching: Flexibility in conceptual memory and pupillary response. *Psychophysiology,**40*(6), 904–913. 14986843 10.1111/1469-8986.00108

[CR25] Walker, M. (1980). *The Lexicon of Comicana*. Comicana. Inc.

[CR26] Weissman, B., Engelen, J., Baas, E., & Cohn, N. (2023). The lexicon of emoji? Conventionality modulates processing of emoji. *Cognitive Science,**47*(4), e13275. 10.1111/cogs.13275 37002916

